# A Study to Estimate the Red Cell Width Distribution and the Mean Platelet Volume in Predicting the 30-Day Mortality in Acute Ischemic Stroke Patients

**DOI:** 10.7759/cureus.57899

**Published:** 2024-04-09

**Authors:** Dhanush Balaji, Abinaya Srinivasa Rangan, Prasanna Karthik Suthakaran, Karpaka Vinayakam Gopalakrishnan, Selva Balaji, Manoj Kumar Sivasamy

**Affiliations:** 1 Internal Medicine, Saveetha Medical College Hospital, Saveetha Institute of Medical and Technical Sciences, Chennai, IND; 2 General Medicine, Saveetha Medical College Hospital, Saveetha Institute of Medical and Technical Sciences, Chennai, IND

**Keywords:** brain ct scan, mean platelet volume, rbc cell width, nihss, acute ischemic stroke

## Abstract

Background

Acute ischemic stroke, a clinical disorder caused by nontraumatic cerebrovascular disease, has an acute onset, frequently causes neurological deficit, and may persist for >24 hours or can be fatal in <24 hours. This study aimed to assess the red cell width distribution (RDW) and the mean platelet volume (MPV) in predicting 30-day mortality in acute ischemic stroke patients. In general, patients with acute ischemic stroke have a rather high mortality rate in the first 30 days due to various complications, but post the 30-day mark, the prognosis is comparatively better.

Material and methods

The present study was conducted on patients with a confirmed diagnosis of acute ischemic stroke based on history, physical examination, CT scan, and/or diffusion-weighted MRI scan performed during the first 24 hours. It was a prospective and cross-sectional study done at Saveetha Medical College over a period of two years. The data was collected by using the intra-hospital network and was analyzed using the IBM SPSS Statistics for Windows, Version 20 (Released 2011; IBM Corp., Armonk, New York, United States).

Results

In the present study, among 100 patients, the mean age was 57.4 ± 13.36 years. About 55% of our subjects were males in our study. The RDW on the 1stday was 14.17 ± 0.708, and it reduced drastically on the 30thday to1st 13.55 ± 1.11, and it was statically significant (p = 0.000). The MPV on day 1 was 11.11 ± 0.969 and, on day 30, was 10.82 ± 0.90; the MPV was reduced considerably on day 30, which was statistically significant (p = 0.000). RDW on the 1st^ ^day was significantly correlated with the MPV and the volume of stroke. The correlation was significant at the 0.01 level (two-tailed). On the 30th^ ^day of acute ischemic stroke patients, the red blood cell (RBC) width was significantly correlated with the MPV. The correlation was significant at the 0.01 level (two-tailed). At the end of 30 days, 10% mortality was observed in the present study. Day 30 saw a significant decrease in the MPV and RDW, particularly in the moderate to severe and severe categories. The National Institutes of Health Stroke Scale (NIHSS) score and the volume of stroke were significantly associated with the 30-day outcome.

Conclusion

The RDW and the MPV are well correlated in predicting the 30-day mortality in acute ischemic stroke patients. This could potentially be used as a significant marker for predicting mortality in stroke patients in the future, but to increase the generalization, further studies need to be carried out at other demographically distinct medical centers.

## Introduction

Acute ischemic stroke, a clinical complaint caused by nontraumatic cerebrovascular complaint, has an acute onset and often results in neurological deficits, which may persist for more than 24 hours or can be fatal in even less than 24 hours [1]. It is one of the leading causes of mortality and morbidity all over the world [2]. Studies have shown that inflammation and oxidative stress may play an important part in the red cell width distribution (RDW) range in ischemic stroke [3]. Oxidative stress is a pathological process brought on by an imbalance in the generation and elimination of reactive oxygen species (ROS). Lipid peroxidation caused by an excess of ROS in the brain tissue can damage the mitochondrial membrane and eventually destroy the respiratory chain. ROS causes neuronal death by damaging mitochondrial DNA, degrading proteins and enzymes in the mitochondria, and destroying the structure and functionality of cell membranes. Additionally, they may impact cerebral blood flow by inducing vasodilation, raising endothelial cell permeability, damaging vascular endothelial cells, raising the permeability of the blood-brain barrier (BBB), and impairing the microcirculation of the brain tissue. These procedures have the potential to harm the brain tissue and aid in the development of stroke [4]. The RDW is a measure of the variation in red blood cell (RBC) sizes, grounded on the mean corpuscular volume (MCV). High RDW position is associated with elevated situations of other seditious labels, viz., C-reactive protein (CRP), erythrocyte sedimentation rate (ESR), and interleukin( IL) situations [5]. Higher RDW values are associated with poorer oxygen supply in tissues, suggesting that decreased oxygenation in the brain may play a direct role in the onset and progression of stroke [6]. Along with high RDW, mean platelet volume (MPV) is also linked with the development of stroke and is explosively associated with all causes of death in cases who are diagnosed with stroke [7]. An increase in MPV causes an aggressive in vivo aggregation in response to adenosine diphosphate (ADP) and collagen. It has now become an independent risk factor for the development of infarction. Acute ischemic stroke patients often have a relatively high death rate in the first 30 days as a result of numerous sequelae; however, after 30 days, the prognosis is significantly better, thus predicting that the mortality can be useful. The present study aimed to estimate the RDW and MPV in prognosticating the 30-day mortality in acute ischemic stroke cases.

## Materials and methods

The present study was conducted at the Department of General Medicine, Saveetha Medical College and Hospital, Chennai, India. Located in Chennai, Tamil Nadu, India, Saveetha Medical College Hospital is a prominent tertiary medical facility. The hospital provides care to a wide range of patients from Chennai and the neighboring areas, thanks to its large campus and array of amenities. With a range of medical specializations, it offers healthcare services to both urban and rural populations. Saveetha Medical College Hospital is a large facility with several buildings devoted to various departments and services. From newborns to the elderly, Saveetha Medical College Hospital's patient population spans a broad demographic range. The study design was prospective and cross-sectional. The study duration was 24 months (January 2021 to December 2022). Study approval was obtained from the institutional ethical committee.

The inclusion criteria included age of more than 18 years, symptoms of stroke less than 24 hours, diagnosis of acute ischemic stroke based on history, physical examination, computed tomography scan and/or diffusion-weighted magnetic resonance imaging (MRI) scan performed during the first 24 hours, and willing to participate in the present study.

The exclusion criteria included computed tomography diagnosis of cerebral hemorrhage, subdural hematoma, intracerebral mass, hemodynamically unstable patients, patients with a previous history of stroke, patients with hematological disorders known to affect platelets and RBCs, patients who are unstable to undergo MRI due to various reasons, and those patients who are not willing to give written informed consent.

Patients with hematological disorders known to affect platelets and RBCs were excluded as their hematological parameters can be altered due to the underlying hematological pathology which can interfere with the results of the study. The study was explained to patients in their local language, and written consent was taken for participation and study. Detailed history taking was done by asking questions such as the onset of muscle weakness, slurring of speech, altered vision, consciousness, and any co-morbidities, and clinical examinations like muscle strength, power, and tone and examinations of the sensory system were undertaken for every patient at admission. All routine investigations like complete hemogram, blood glucose, liver and kidney function test, lipid profile, electrolytes, urine exam, and electrocardiography were done. A computed tomography scan of the brain was done in all patients after hospitalization. All patients received standard care. The outcome in stroke patients was assessed in terms of the 30-mortality since the stroke episode. Follow-up was kept till three months. Red cell width distribution and MPV values at Day 1 and day 30 were noted. Statistical analysis was done using the IBM SPSS Statistics for Windows, Version 20 (Released 2011; IBM Corp., Armonk, New York, United States). Students t-test, Chi-square test, and frequency distribution were used. Pearson’s test was used to analyze correlation. Analysis of variance (ANOVA) was used for multivariate analysis. A prospective design was used for the study, which reduced recall bias and enabled real-time data gathering, improving the dependability of the results. Every patient was given a thorough history and clinical examination upon admission, and regular investigations were carried out in accordance with established protocols. The gathered data is more reliable and has less fluctuation because of this uniform procedure. RDW and MPV should be measured according to established laboratory procedures to guarantee measurement accuracy and consistency. To preserve instrument performance, calibrate the hematology analyzer on a regular basis in accordance with the manufacturer's instructions. As a normal procedure, assess stroke severity at baseline and follow-up intervals using validated modalities, such as the National Institutes of Health Stroke Scale (NIHSS). The study allowed for the evaluation of results over time by including a 30-day follow-up period following the stroke episode. This longitudinal method sheds light on how the illness develops and how well therapies work.

## Results

The objective of this research was to determine whether the MPV and the RDW might be used as biomarkers to predict 30-day death in patients suffering from acute ischemic stroke. In the present study, among 100 patients, the mean age was 57.4 ± 13.36 years. In our study, 55% of our subjects were males. The mean age of the male subjects in our study was 54. 45 ± 13.2, and the mean age of females was 61.00 ± 12.77, and it is statistically significant (p = 0.014). The maximum number of our patients was between 41 and 60 years of age (44%), followed by above 60 years of age (42%), and only 14 were between 20 and 40 years of age. The most common co-morbidities noted were diabetes mellitus (46%), followed by hypertension (27%), sustained hypertension (21%), and chronic kidney diseases (6%). Co-morbidities were assessed by laboratory tests like fasting blood glucose, post-prandial blood glucose, and HbA1c to assess for diabetes. A renal function test was done to rule out any chronic kidney diseases, and the blood pressure was measured using the sphygmomanometer to assess whether the patient was in a hypertensive state. These co-morbidities are known risk factors for developing acute ischemic stroke and can potentially have a worse prognosis.

The RDW on the 1st day was 14.17 ± 0.708, and it reduced drastically on the 30th day to 13.55 ± 1.11, which was statically significant (p = 0.000). The MPV on day 1 was 11.11 ± 0.969 and, on day 30, was 10.82 ± 0.90. The MPV was reduced considerably on day 30, and it was statistically significant (p = 0.000). On the first day, 20 were in the minor stroke category, 56 were in moderate, 19 were in moderate to severe, and five were in severe stroke. At the end of the 30th day, the subjects in the minor category increased to 32, 54 became moderate in severity, 11 were in moderate to severe, and only three were in severe stroke. Thus, the overall severity reduced after 30 days. The RDW in minor, moderate, moderate to severe, and severe stroke categories was 13.73 ± 0.77, 13.93 ± 0.355, 14.94 ± 0.313, and 15.64 ± 0.320, respectively. However, on day 30, RDW was reduced in all categories. It was 12. 73 ± 1.09 in mild and 13.69 ±0.787 in moderate category, which was statistically significant. Though the RDW didn’t show a significant difference, the RDW was decreased in the moderate to severe and severe categories. The inter-group comparison showed a statistically significant difference (p = 0.000).

**Figure 1:**
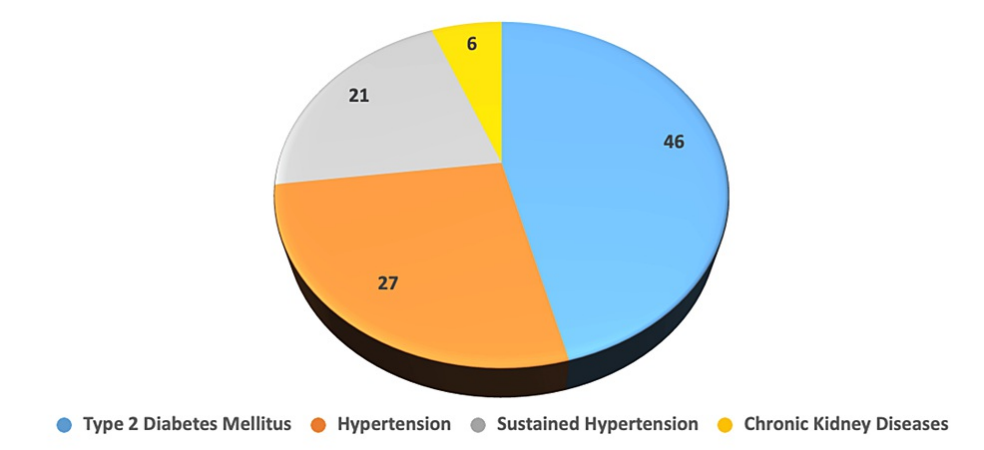
Prevalence of various co-morbidities

Comparing the MPV in different severities of stroke, the score decreased on day 30 in the moderate and moderate to severe categories, and the score is increased slightly in the mild and severe categories. The inter-group comparison showed a statistically significant difference (p = 0.000). The RDW on the 1st day was significantly correlated with the MPV and volume of stroke. The correlation was significant at the 0.01 level (two-tailed). On the 30th day of acute ischemic stroke patients, red cell width was significantly correlated with MPV. The correlation was significant at the 0.01 level (two-tailed). At the end of 30 days, 10% mortality was observed in the present study. The NIHSS score and volume of stroke were significantly associated with the 30-day outcome (Tables 1-5) (Figure 1).

**Table 1:** Comparison and distribution of the mean age and gender ns: Not statistically significant

Demographic parameter	Male (mean ± SD) (n = 55)	Female (mean ± SD) (n = 45)	t values	p-values
Age	54.45 ± 13.22	61.00 ± 12.77	-2.500	0.014**
Distribution of age	Male (%)	Female (%)	Total (%)	
20-40	10 (71.4)	04 (28.6)	14 (100)	0.285 (ns)
41-60	25 (56.8)	19 (43.2)	44 (100)	
>60	20 (47.6)	22 (52.4)	42 (100)	

**Table 2:** Comparison of red cell width distribution, mean platelet volume, and the NIHSS score on the 1st and 30th day NIHSS: National Institutes of Health Stroke Scale

Parameter	1st day	30th day	Mean difference	t values	p-values
Cell width distribution	14.17 ± 0.708	13.55 ± 1.11	0.619	5.884	0.000***
Mean platelet volume	11.11 ± 0.969	10.82 ± 0.90	0.299	4.542	0.000***
Interpretation of NIHSS Score	Severity of stroke on the 1st day	Severity of stroke on the 30th day			
Minor stroke	20	32			
Moderate stroke	56	54			
Moderate to severe stroke	19	11			
Severe stroke	05	03			
Total	100	100			

**Table 3:** Comparison of the red cell width distribution and mean platelet volume in different severities of stroke based on the NIHSS score between the 1st and 30th day NIHSS: National Institutes of Health Stroke Scale; ns: not statistically significant

Severity of stroke based on the NIHSS score	Red cell width distribution on 1st day	Red cell width distribution on 30th day	t values	p–values	Mean platelet volume on 1stday	Mean platelet volume on 30th day	t values	p–values
Minor stroke	13.73 ± 0.77	12.73 ± 1.09	3.620	0.000***	9.73 ± 0.88	9.94 ± 0.782	-0.896	0.375 (ns)
Moderate stroke	13.93 ± 0.355	13.69 ± 0.787	2.037	0.04**	11.13 ± 0.361	10.99 ± 0.384	-1.34	0.189 (ns)
Moderate-severe stroke	14.94 ± 0.313	14.73 ± 0.420	1.568	0.128 (ns)	12.12 ± 0.214	12.04 ± 0.301	0.801	0.430 (ns)
Severe stroke	15.64 ± 0.320	15.53 ± 0.288	1.27	0.14 (ns)	12.61 ± 0.192	12.63 ± 0.152	0.355	0.735 (ns)

**Table 4:** Pearson’s correlation test for the 1st and 30th day parameters RBC: Red blood cell; **: correlation is significant at the 0.01 level (two-tailed)

	RBC width 1st day	Mean platelet volume 1st day
RBC width 1st day	1	.546^**^
Mean platelet volume 1st day	.546^**^	1
	Mean platelet volume 30th day	RBC width 30th day
Mean platelet volume 30thday	1	.428^**^
RBC width 30th day	.428^**^	1

**Table 5:** Comparison of parameters among survivor and death patients *p < 0.05-**p < 0.001: Statistically significant; ns: not statistically significant

Parameter	Survivor patients N = 90		Death patients N = 10	
	Parameter on the1stday	Parameter on the 30thday	Parameter on the 1st day	Parameter on the 30th day
RBC width	14.06 ± 0.65	13.2 ± 0.28	15.07 ± .57	15.56 ± 1.17
	t value: 7.826; p = 0.00***		t value: -0.996; p = 0.345 (ns)	
Mean platelet volume	10.95 ± .94	10.55 ± .782	11.90 ± .270	11.71 ± .166
	t value: 4.920; p = 0.000**		t value: 2.237; p = 0.052**	

## Discussion

Acute stroke is one frequent cause of exigency admission. Stroke, being the complaint of the senior, is associated with high morbidity and mortality rates. Stroke is the alternate most common cause of mortality and the third most common cause of disability worldwide. Encyclopedically, 68 of all strokes are ischemic, and 32 are hemorrhagic [8]. In India, the frequency of stroke was 147 per lakh, and the periodic prevalence rate was 36 per lakh. The overall frequency of stroke ranges from 147 per lakh to 922 per lakh according to various studies [7-9]. A stroke is defined as an unforeseen onset of a neurological deficiency attributable to a focal vascular cause. It's one of the leading causes of mortality and morbidity all over the world. Stroke can be classified into ischemic and hemorrhagic stroke. Around 85 of all strokes are ischemic, and the remaining is hemorrhagic. Dislocation of blood force to the brain causes ischemic stroke. The present study demonstrated the correlation of the volume of stroke by MRI brain, RDW, MPV, and NIHSS scoring in prognosticating 30-day mortality in acute ischemic stroke cases. In a recent study, the relationship between RDW and stroke was investigated, and it was found that increased RDW was associated with a worse prognosis and mortality [10].

In the present study, the mean age was 57.4 with the minimum age of 24 years and the maximum of 83 years. The maximum number of our patients was between 41 and 60 years of age, and there were about 44 subjects. Around 42 subjects were above 60 years, and only 14 were between 20 and 40 years of age. The gender distribution in our study shows predominantly males. They were around 55% of our subjects. The mean age of the male subjects in our study was 54.45 ± 13.2, and the mean age of females was 61.00 ± 12.77, which is statistically significant (p = 0.014). The most common co-morbidities among our subjects were diabetes mellitus which was around 46%. About 27 of our subjects had hypertension, and 21% had sustained hypertension. About 6% had chronic kidney disease as a complication. The NIHSS was originally designed for prospective scoring, but it has been used for the retrospective assessment of initial stroke severity [11-12]. The NIHSS score on day 1 was 9.70 ± 5.22, and on day 30, the score was 7.77 ± 5.25. This is statistically significant (p = 0.000). The RDW and MPV were shown to be significantly correlated on the 1st day of acute ischemic stroke (p < 0.01; two-tailed). Similarly, there was a strong two-tailed correlation between the RDW and MPV on the 30th day following an acute ischemic stroke (p < 0.01).

The RDW is a parameter that reflects the heterogeneity of the RBC volume. Jia et al. [12] have found that the levels of RDW in these patients with acute ischemic stroke were higher than those with no stroke. Likewise, Soderholm et al. [13] found that high levels of RDW could increase the risk of stroke or CI in a population-based cohort study. We have observed that the RDW distribution on the 1st day was 14.17 ± 0.708, and it reduced drastically on the 30th day, which was around 3. 55 ± 1.11, and it is highly statically significant (p = 0.000). The RDW on day 1, in the minor, moderate, moderate to severe, and severe stroke category was 13.73 ± 0.77, 13.93 ± 0.355, 14.94 ± 0.313, and 15.64 ± 0.320, respectively. However, on day 30, the RDW was reduced in all categories. It was 12.73 ± 1.09 in the mild and 13.69 ± 0.787 in the moderate category, which was statistically significant. Though the exact mechanism is not known, inflammation antioxidative stress (OS) may play an important role in RDW in ischemic stroke. RDW values were related to the levels of oxidation and antioxidants, which were related to the severity of ischemic stroke. We have found that the NIHSS in our study is significantly correlated with RDW. Earlier studies have found a similar significant association between NIHSS score and RDW value on emergency admission [14-16]. Turcato et al. [17] reported that the RDW (14.0% vs 13.6%; p < 0.001) and the NIHSS score (12 vs 4; p < 0. 001) upon admission were found to be significantly increased in patients with acute ischemic stroke with unfavorable outcome compared to those with favorable outcomes. So, based on our findings the RDW can be used to predict the prognosis of acute ischemic stroke patients. While high RDW levels don't directly affect the NIHSS score, they may be a sign of an underlying inflammation and a poor prognosis for stroke patients. Alternatively, the two metrics can offer supplementary insights on the patient's general health and prognosis. Also blood cell abnormalities may be caused by underlying medical disorders or hereditary factors rather than by sex. Additionally, a statistically significant link was found between the RDW and the volume of stroke and the NIHSS score; however, more research is required to ascertain the degree of this correlation.

In our study, the mean value of MPV at day 1 was 11.11 ± 0.969 and, at day 30, was 10.82 ± 0.90. This shows that the MPV has reduced considerably in day 30, and it was statistically significant (p = 0.000). Additionally, depending on the NIHSS score, there is a link between the MPV and the severity of stroke. In particular, on the 1st and 30th day, there was a significant difference in the MPV between the mild, moderate, moderate to severe, and severe stroke severity categories. Previous studies have demonstrated higher levels of MPV in patients with acute ischemic stroke than in control subjects [18-20]. Pikija et al. have reported that higher MPV was independently associated with larger infarct size, and higher risk of early and mid-term death after stroke [21]. It was observed that the MPV was observed as independent risk factor for ischemic stroke and correlated with poorer outcome [22]. Increased RDW values are associated with poorer oxygen supply in tissues, suggesting that decreased oxygenation in the brain may play a direct role in the onset and progression of stroke. 

In our study, we have observed that the MPV on day 1 in minor stroke was 9.73 ± 0.88, moderate was 11.13 ± 0.361, moderate to severe was 12.12 ± 0.214, and severe category was 12.61 ± 0. 192. On day 30, the score decreased in moderate and moderate to severe category, which was around 10.99 ± 0.384 and 12.04 ± 0.301, respectively. It has been found that the NIHSS score in our study is significantly correlated with the MPV. In patients with high risk factors for stroke, regular evaluation of renal function could reduce the risk of stroke as well as complication and mortality after stroke [23]. The specific causes for the adverse outcome and whether a more aggressive therapeutic approach can improve the prognosis of these patients should be assessed by future studies. Because only one center was used for the study, it's possible that the results cannot be applied to other groups or circumstances. Results may differ between healthcare facilities due to differences in patient demographics, clinical procedures, and available resources. Selection bias may be unintentionally introduced by the study's inclusion criteria, since individuals with particular traits or co-occurring conditions may make them more likely to match the requirements. The study population's representativeness may be impacted; for instance, individuals with hemodynamic instability or hematological illnesses that are known to impact RBCs and platelets are excluded. Future directions for investigating the relationship between the RDW, MPV, and stroke may involve large prospective studies, longitudinal studies, and genetic studies that can shed light on the genetic basis of these traits and their role in the pathophysiology of stroke. Large prospective studies can involve a diverse population of stroke patients and validate the findings observed.

## Conclusions

We have observed that the NIHSS score is significantly correlated with RBC width and the MPV. In high-risk individuals in particular, this can be utilized as a biomarker to determine prognosis and enable more intensive treatment. The RDW and MPV are well correlated in predicting the 30-day mortality in acute ischemic stroke patients.
